# Tacrolimus ameliorates functional disturbances and oxidative stress in isoproterenol-induced myocardial infarction

**DOI:** 10.1186/s40199-014-0068-3

**Published:** 2014-10-14

**Authors:** Arash Khorrami, Mojtaba Hammami, Mehraveh Garjani, Nasrin Maleki-Dizaji, Alireza Garjani

**Affiliations:** Department of Pharmacology and Toxicology, Faculty of Pharmacy, Tabriz University of Medical Sciences, Tabriz, Iran; Student Research Committee, Tabriz University of Medical Sciences, Tabriz, Iran

**Keywords:** Tacrolimus, Myocardial infarction, Isoproterenol, Electrocardiography

## Abstract

**Background:**

The inflammatory responses play a major role in the pathogenesis of acute myocardial infarction (MI). Early inhibition of inflammation may improve post MI cardiac function. The aim of this study was to investigate the effects of tacrolimus on cardiac function, hemodynamic parameters as well as histopathologic and electrocardiographic changes in isoproterenol-induced myocardial infarction.

**Methods:**

Male Wistar rats were randomly divided into six groups of control, isoproterenol alone, tacrolimus alone, and isoproterenol plus tacrolimus (0.5, 1 and 2 mg/kg). Isoproterenol (100 mg/kg) was injected subcutaneously for two consecutive days to induce myocardial infarction, and simultaneously tacrolimus was administered orally twice a day for three days.

**Results and conclusions:**

Administration of isoproterenol resulted in myocardial edema and necrosis as well as a marked reduction in the left ventricular systolic pressure (LVSP), left ventricular contractility (LVdP/dt_max_) and relaxation (LVdP/dt_min_) along with a severe elevation in left ventricular end-diastolic pressure (LVEDP). Isoproterenol also elevated the ST-segment and suppressed the R-amplitude and R-R interval on ECG. It was found that all doses of tacrolimus could amend the ECG pattern and ameliorated the isoproterenol induced disturbances in cardiac function. Acute and short term treatment with tacrolimus at dose of 2 mg/kg significantly (P < 0.001) improved LVdP/dt_max_ from 2712 ± 82 in myocardial infarcted rats to 4592 ± 149 mmHg/sec. Similarly, tacrolimus lowered LVEDP from 17.6 ± 0.68 in MI group to the value of 5.6 ± 0.22 mmHg (P < 0.001). Furthermore, tacrolimus was found to reduce malondialdehyde concentration in serum and myocardium by 50-70% (P < 0.001).

The results of this study showed that acute treatment with tacrolimus, coincided with the occurrence of myocardial infarction, strongly protected the myocardium against the isoproterenol-induced myocardial infarction; where this might be due to the anti-inflammatory properties of tacrolimus.

## Background

Cardiovascular disease (CVD), predominantly myocardial infarction, is the leading cause of death worldwide [1,2]. Reductions in the myocardial blood flow due to the coronary artery occlusion can considerably compromise the energy metabolism. Coronary artery occlusion, only as short as 5 minutes, can lead to oxidative stress and then functional abnormalities of the myocardium that persist up to 24–48 hours after reperfusion [3-5].

The existing therapeutic approaches are designed to reduce the myocardial necrosis and also amendment of cardiac function after a myocardial infarction. Several studies have shown that consecutive vigorous inflammatory and immune reactions increases the generation of cytokines and oxidative stress, recruits inflammatory and T cells, and initiates the complement cascade [6,7]. The inflammatory responses induced by myocardial ischemia play a major role in the pathogenesis of acute myocardial infarction (AMI). In addition, recent studies have demonstrated that myocardial necrosis and subsequent oxidative stress trigger a cytokine cascade initiated by Tumor Necrosis Factor (TNF)-α. These inflammatory responses are found to exacerbate myocardial injury and remodeling after AMI [1,6,8], which lead to a progressive and irreversible myocardial damage. One of the principal interventions that exist for AMI is early inhibition of the inflammatory cycle and thus achieves a better recovery of the myocardium.

Tacrolimus (FK506) is a macrolide produced by the bacteria *Streptomyces tsukubaensis*. It is an immunosuppressant mainly used in organ transplantation in order to reduce the risk of graft rejection. Activation of T cells by allogenic antigens in transplanted tissue increases intracellular calcium concentration that contributes to the calcium-calmodulin complex formation. Calcium-calmodulin complex, in turn, activates calcineurin, a crucial phosphatase for T cell signaling [9,10]. Tacrolimus binding to the FK506 binding protein (FKBP), a respective cytoplasmic receptor, inactivates calcineurin, thereby the inflammatory response. FKBP is present in most tissues, including the myocardium. In addition to the anti-inflammatory effects of FK506-FKBP complex, it plays a critical role in modulating myocardial calcium homeostasis and rhythm regulation by stabilizing Ryanodine receptors [7,11]. The complex of FK506-FKBP inhibits the phosphatase activity of calcineurin and the calcium-dependent inflammatory responses. Moreover, some experimental studies have revealed that tacrolimus can effectively inhibit MAPK and PI3K-Akt signaling pathways in various inflammatory diseases [7].

Isoproterenol (ISO) is a synthetic beta-adernoceptor agonist and its subcutaneous injection induces irreversible cellular damage and ultimately myocardial infarction in rats. The acute hemodynamic and electrocardiographic changes in isoproterenol induced myocardial infarction resemble closely to those occurring in patients with myocardial infarction [12]. Therefore, the rat model of isoproterenol induced myocardial infarction offers a reliable non-invasive technique for studying the effects of various potentially cardioprotective agents [13].

The aim of the present study was to investigate the potential cardioprotective effects of tacrolimus on acute heart remodeling, hemodynamic, electrocardiographic, and biochemical changes in isoproterenol-induced myocardial infarction in rats.

## Materials and methods

### Animals

Male albino Wistar rats weighing between 260 and 280 g were used in the present study. The animals were housed in standard polypropylene cages (six per cage) under a 12/12 h light/dark cycle at a constant temperature of 20 ± 1.8°C and ambient humidity of 50 ± 10%. The rats were given food and water freely. This study was performed in accordance with the Guide for the Care and Use of Laboratory Animals of Tabriz University of Medical Sciences, Tabriz-Iran (National Institutes of Health publication No 85–23, revised 1985).

### Induction of acute myocardial infarction

Isoproterenol (Sigma Co; USA) was dissolved in normal saline and injected subcutaneously to rats (100 mg/kg) for two consecutive days at an interval of 24 hours to induce acute myocardial infarction. Animals were sacrificed 48 hours after the first injection of isoproterenol [13].

### Experimental protocol

The animals were randomly divided into six groups consisting of six rats each. Rats in group1 (normal control) received a subcutaneous injection of normal saline (0.5 ml) and were left untreated for the entire experimental period. Rats in group 2 (sham) were received an oral administration of tacrolimus (2 mg/kg; twice daily) for two consecutive days and were subcutaneously injected with normal saline. To induce myocardial infarction, rats in group 3 (MI control; ISO) were subcutaneously (SC) injected with isoproterenol (100 mg/kg) dissolved in normal saline as a vehicle (0.5 ml) for two consecutive days at an interval of 24 h. Rats in groups 4 to 6 were orally treated with tacrolimus at 0.5, 1 and 2 mg/kg twice daily. The treatment was started immediately prior to induction of MI (SC injection of isoproterenol) for the ensuing 2 days.

### Hemodynamic measurements

About 24 to 48 hours after the last administration of drug, the animals were anesthetized by the intraperitoneal injection of a mixture of ketamine (50 mg/kg), xylazine (30 mg/kg), and acepromazine (20 mg/kg). When rats did not respond to noxious stimuli, a standard limb lead (II) observed constantly during the experimental period and any changes in ECG pattern were documented. To observe the hemodynamic parameters, a ventral midline skin incision was made from the lower mandible posteriorly to the sternum (~3 cm) and the left common carotid artery was isolated. The artery was temporary occluded toward the heart using a dieffenbach serrefines clamp (Elcon; Germany). Vannas micro iris scissors (Medi Plus; USA) was used to make a tiny incision on the carotid artery. A polyethylene cannula (Protex; OD 0.98 mm, ID 0.58 mm), coupled to a pressure transducer (Powerlab system; AD Instruments, Australia), was inserted into the artery for the recording systemic arterial blood pressure. The mean arterial blood pressure was calculated from the systolic and diastolic blood pressure traces. To assess the cardiac left ventricular function, a Mikro-Tip® catheter transducer (Millar Instruments, INC) was inserted to the lumen of the left ventricle. This helped to measure the left ventricular systolic pressure (LVSP), left ventricular end-diastolic pressure (LVEDP), maximum and minimum rates of developed left ventricular pressure (LVdP/dt_max_ and LVdP/dt_min_ respectively) and the rate of pressure change at a fixed left ventricular pressure (LVdP/dt/P). All the parameters were continuously recorded using a Powerlab system (AD Instruments, Australia) [14].

### Tissue weights

Subsequent to hemodynamic measurements, the animals were euthanized, the hearts were removed, weighed, and histological examinations were done. The wet heart weight to body weight ratio was calculated to assess the degree of myocardial weight gain.

### Determination of lipid peroxidation in serum and myocardium

Malondialdehyde (MDA), a thiobarbiturate reactive substance, was measured as a marker for oxidative stress in serum and myocardial homogenates using a method prescribed by Satoh [15]. To prepare the homogenates, another set of experiment (n = 6) including all groups was repeated and 500 mg of heart tissues from apex were homogenized in a ratio of 1:10 in 0.15% KCl by means of a homogenizer. Consequently the homogenates were centrifuged at 4 000 rpm at 4°C for 10 min. Then 3 mL of phosphoric acid and 1 mL of TBA 0.67% were added to 250 μL of supernatant of each homogenate to analyze the MDA level in tissue samples. After addition of 3 mL of n-Butanol to each sample, they heated in a water bath for 40 min and the absorbance of MDA was measured spectrophotometrically. The lipid peroxidation level expressed as nanomole MDA production per gram myocardium and nonomole per milliliter serum.

### Histopathological examination

The hearts were fixed in 10% buffered formalin. The heart tissues were embedded in paraffin, sectioned at 5 μm, and stained with hematoxylin and eosin (H&E) for histologic evaluation and observed microscopically. Myocardial necrosis and edematous were evaluated in each of the four quadrants of the left ventricular cross section in each heart in the zone lying within 1.5 mm of the ventricular endocardium, using a morphometric point-counting procedure [16]. Two persons graded the histopathological changes as 1, 2, 3, and 4 for low, moderate (focal myocyte damage or small multifocal degeneration with slight degree of inflammation), high (extensive myofibrillar degeneration and/or diffuse inflammatory process), and intensive (necrosis with diffuse inflammatory process) pathological changes, respectively.

### Measurements of serum TNF-α level by ELISA

The serum level of TNF-α was determined using an enzyme-linked immunosorbent assay (ELISA) (Rat TNF-α, IBL, Hamburg, Germany) according to the manufacture’s instruction. Briefly, the samples were homogenized or mixed in an ice-cold solution containing 50 mM Tris–HCl, 150 mM NaCl, 5 mM Sodium Pyrophosphate (NaPPi), 50 mM NaF, 1 mM EDTA, 1 mM.

dithiothreitol (DTT), 0.1%SDS (w/v), 1% TXT-100 (v/v), and protease inhibitor cocktail (Roche, Mannheim, Germany). The samples were then centrifuged twice at 10,000 (rpm) for 10 min at 4°C. The resulting supernatants were used for assay. The concentration of the cytokines was expressed as pg/ml of serum.

### Statistics

Data were presented as mean ± SEM. One-way ANOVA was used to make comparisons between the groups. If the ANOVA analysis indicated significant differences, Student–Newman–Keuls post test was employed to compare the mean values between the treatment groups and the control. Differences between the groups were considered significant if P < 0.05.

## Results

### Effects of tacrolimus on electrocardiogram

**Figure 1:**
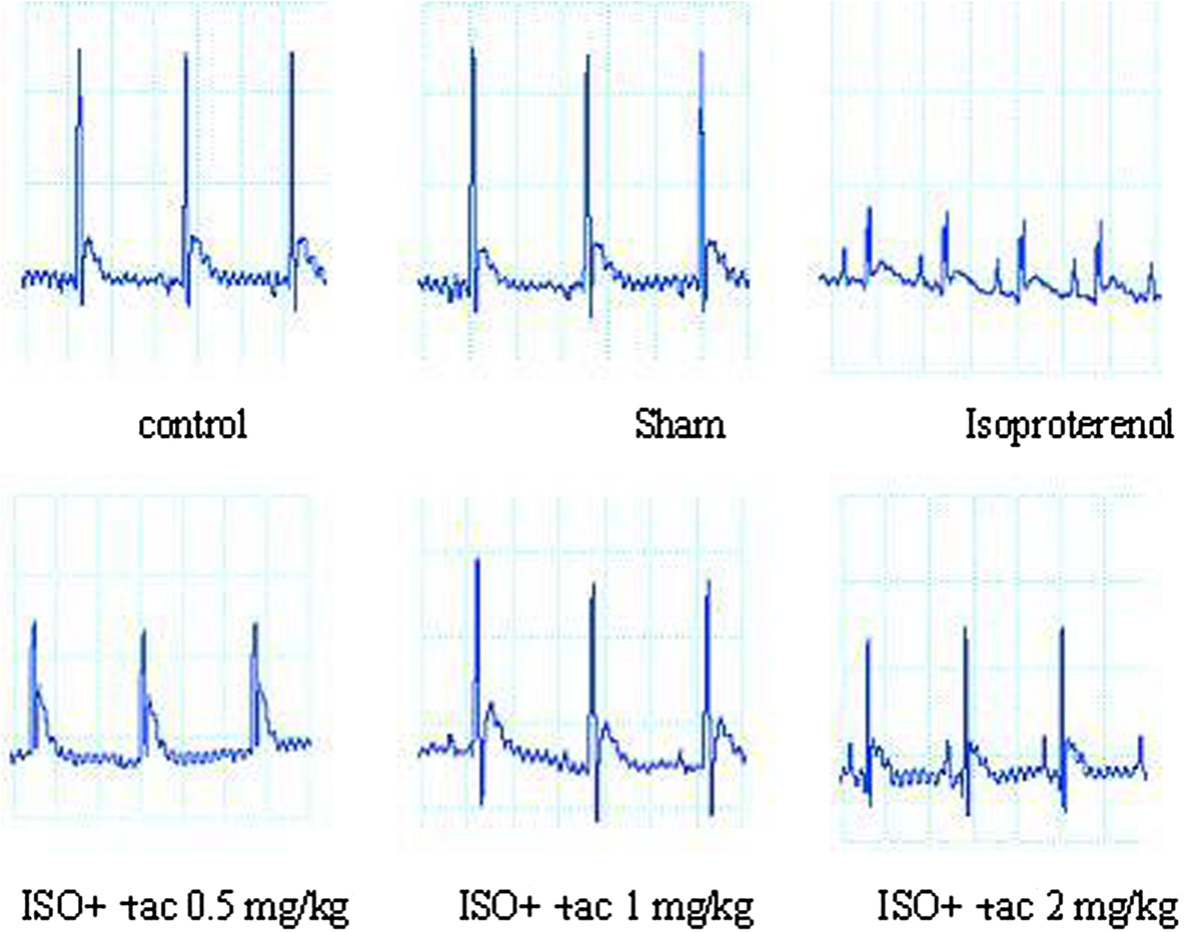
**Effect of tacrolimus on electrocardiographic patterns and changes (recorded from limb lead II) in normal control**, **tacrolimus alone treated group (Sham)**, **isoproterenol alone injected (ISO), and rats treated with tacrolimus (Tac**: **0.5**, **1**, **2 mg**/**kg).**

**Figure 2:**
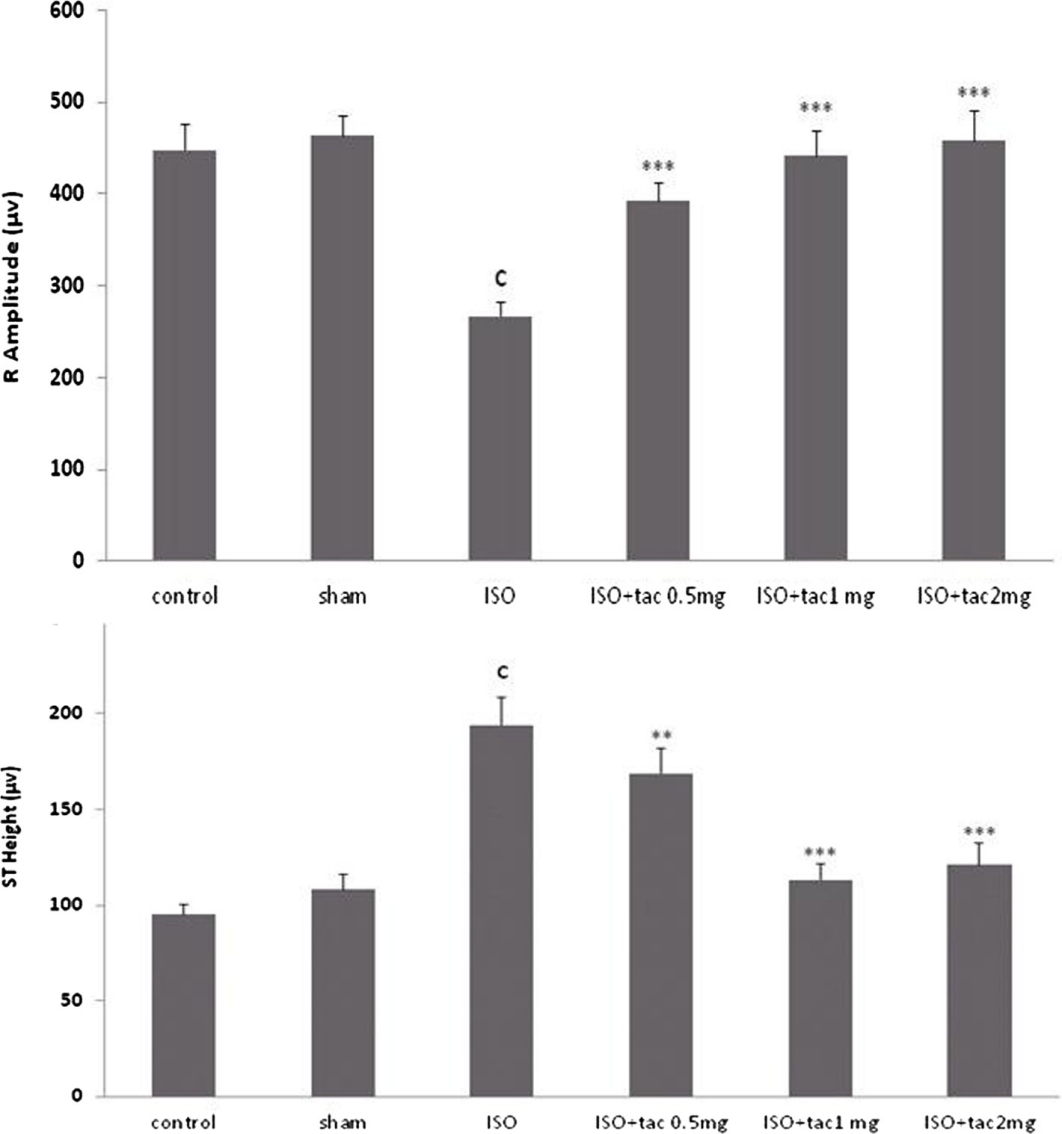
**Effects of oral administration of tacrolimus (tac) on ST segment and R**-**amplitude (recorded from limb lead II).** ^c^P < 0.001 compared to normal controls. **P < 0.01 and ***P < 0.001 as compared to the ISO-treated group. Data are reported as mean ± SEM (n = 6).

The Lead II electrocardiograms obtained from animals were shown in Figure 1. The rats were received tacrolimus alone (sham) showed normal patterns of ECG, while the rats treated with isoproterenol alone (MI group) demonstrated significant changes in ECG pattern. The changes included a marked elevation of ST segment from 95 ± 5.2 μv in control group to 194 ± 14.2 μv in ISO-treated group (P < 0.001), and a reduction in R wave amplitude from 448 ± 27 μv in control group to 267 ± 15 μv in ISO-treated group (P < 0.001) which both are indicative of myocardial infarction (Figure 2). Oral treatment with tacrolimus at doses of 0.5, 1 and 2 mg/kg significantly suppressed the isoproterenol-induced ST-segment elevation from 194 ± 14.2 μv in ISO-treated group to 169 ± 12.7, 113 ± 8.4 and 121 ± 11 μv in tacrolimus-treated groups, respectively.

Furthermore, treatment with all doses of tacrolimus resulted in a significant increase in the R-amplitude (P < 0.001) as compared to the rats treated with isoproterenol alone (Figure 2).

### Effects of tacrolimus on hemodynamic responses

The mean arterial pressure (MAP) was significantly decreased from 79.5 ± 1.78 mmHg in control group to 37 ± 1 mmHg in isoproterenol-treated group (P < 0.001; Table 1). There was a significant increase in the mean arterial pressure to the value of 52 ± 1.12, 79 ± 1.86 and 78.2 ± 2 mmHg, respectively, after administration of 0.5, 1 and 2 mg/kg tacrolimus.

**Table 1:** **Effects of short term oral administration of tacrolimus (tac) on hemodynamic parameters in normal and isoproterenol (ISO) induced myocardial infarction rats**

**Groups**	**MAP ± ** **SEM**	**HR ± ** **SEM**	**LVSP ± ** **SEM**	**LVEDP ± ** **SEM**	**LVdp/** **dt/** **p ± ** **SEM**
	**(mmHg)**	**(bpm)**	**(mmHg)**	**(mmHg)**	**(1/** **sec)**
**Control**	79.5 ± 1.78	197.8 ± 3.05	112.8 ± 2.71	6.3 ± 0.21	85.2 ± 1.49
**Sham**	80.4 ± 2.52	216.4 ± 6.21	117.6 ± 4.03	5.4 ± 0.36	85.6 ± 1.52
**ISO**	37 ± 1.05^c^	290 ± 7.9^c^	72.8 ± 1.389^c^	17.6 ± 0.68^c^	53.4 ± 1.07^a^
**ISO + ** **tac 0.5 mg**	52 ± 1.12*	247 ± 7.35*	83.4 ± 1.57	10.2 ± 0.45***	65 ± 1.96*
**ISO + ** **tac 1 mg**	79.8 ± 1.86***	200.2 ± 4.84***	120.6 ± 3.76***	6 ± 0.24***	86.6 ± 2.07***
**ISO + ** **tac 2 mg**	78.2 ± 2.06***	212.8 ± 5.43***	117.8 ± 3.24***	5.6 ± 0.22***	83.4 ± 1.95***

^a^P < 0.05, ^c^P < 0.001 compared to normal control group. *P < 0.05 and ***P < 0.001 as compared to the ISO-treated group. Mean arterial pressure (MAP), heart rate (HR), left ventricular systolic pressure (LVSP), left ventricular end diastolic pressure (LVEDP), left ventricular pressure changes at a fixed pressure (LV dP/dt/P). Data are shown as mean ± SEM (n = 6).

The intraventricular pressure was measured to determine the degree of left ventricular response to isoproterenol. Isoproterenol reduced the LVSP from 112.8 ± 2.71 mmHg in the control group to 72.8 ± 1.38 mmHg (P < 0.001). Tacrolimus was found to increase the LVSP toward the normal value in a dose-dependent manner.

Furthermore, isoproterenol treatment leads to almost a three-fold elevation in LVEDP, which was indicative of left ventricular dysfunction. All three doses of tacrolimus remarkably (P < 0.01) improved the left ventricular function by lowering LVEDP from 17.6 ± 0.68 mmHg in isoproterenol-treated rats to 10.2 ± 0.45, 6 ± 0.24 and 5.6 ± 0.22, respectively (Table 1).

**Figure 3:**
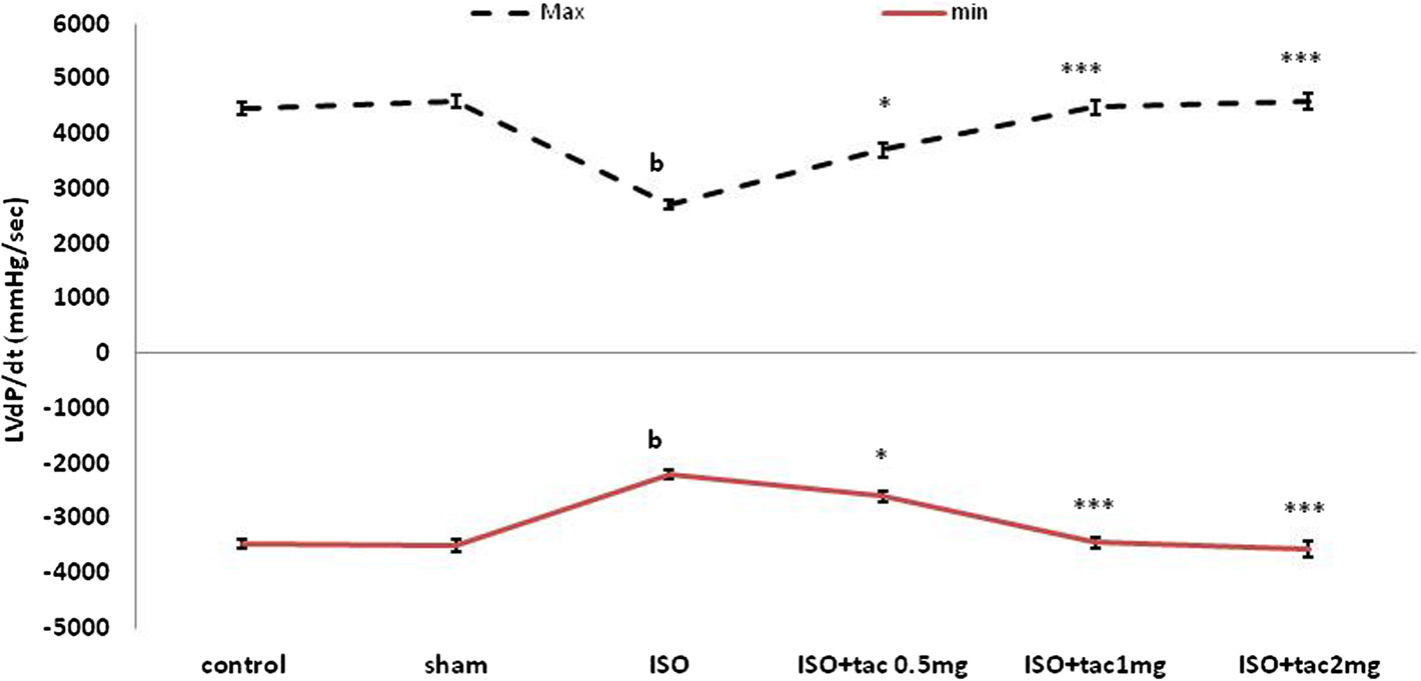
**The effect of oral administration of tacrolimus** **(tac)** **on the minimum and maximum left ventricular pressure** **(LVdP/** **dt**_**Max**_ **, LVdP/** **dt**_**Min**_ **).** ^c^P < 0.001 as compared to the normal control group, *P < 0.05 and ***P < 0.001 as compared to the ISO-treated group. Data are reported as mean ± SEM (n = 6).

Compared with the control group, the rats with left ventricular dysfunction (MI group) demonstrated a reduction in the value of left ventricular maximal and minimal rates of pressure (LV dP/dt_max_; LV dP/dt_min_; P < 0.01; Figure 3) as well as a lower rate of pressure change at a fixed ventricular pressure (LV dP/dt/P; P < 0.05; Table 1). The mentioned indices of myocardial contractility, along with LVSP, were considerably improved by administration of all three doses of tacrolimus (Figure 3). In this regard, tacrolimus at doses of 0.5, 1 and 2 mg/kg significantly increased LV dP/dt_Max_ to 3702 ± 308 (P < 0.05), 4484 ± 318 (P < 0.001) and 4592 ± 333 mmHg/s (P < 0.001), respectively, from 2712 ± 181 mmHg/s in isoproterenol alone treated group. Tacrolimus had a similar effect on LV dP/dt_Min_.

### Effects of tacrolimus on the heart weight to body weight ratio

**Figure 4:**
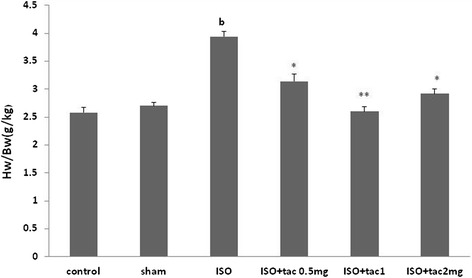
**Effects of isoproterenol** **(ISO)** **injection as well as tacrolimus** **(tac)** **on heart weight (** **HW)** **to body weight (** **BW)** **ratio as an index of tissue edemathose in different groups.** ^b^P < 0.01 compared to respective group in healthy animals, *P < 0.05 and **P < 0.01 as compared to isoproterenol (ISO) treated group. Data are reported as mean ± SEM (n = 6).

The heart weight to body weight ratio (mg/kg) was determined to evaluate the amount of heart weight gain developed by the injection of isoproterenol (Figure 4). Isoproterenol injection significantly increased the ratio from 2.58 ± 0.22 in control group to 3.94 ± 0.14 (P < 0.01). Acute treatment with 1 mg/kg of tacrolimus resulted in a substantial (P < 0.01) reduction in the heart to body weight ratio in comparison to the isoproterenol alone treated rats.

### Effects of tacrolimus on lipid peroxidation

To determine the lipid peroxidation, MDA levels were measured in serum and myocardial homogenates. Both serum and heart MDA levels were considerably increased (P < 0.001) in isoproterenol alone treated rats in comparison with normal control group (Table 2). However, treatment with tacrolimus significantly suppressed the rise of MDA levels of serum and myocardium (P < 0.001; Table 2).

**Table 2:** **The effects of acute treatment with tacrolimus (tac) on malondialdehyde (MDA) levels in the serum and heart tissue of control and rats with myocardial infarction**

**Groups** ** (n = ** **6)**	**MDA (** **nmol/** **mL serum)**	**MDA (** **nmol/** **g tissue)**
**Control**	5.4 ± 0.48	2.7 ± 0.08
**sham**	4.9 ± 0.3	2.4 ± 0.09
**Isoproterenol** ** (Iso)**	13.7 ± 0.2^a^	5.78 ± 0.17^c^
**Iso** ** + tac** ** (0.5 mg/** **kg)**	7.8 ± 1.7***	4.54 ± 0.12**
**Iso + ** **tac** ** (1 mg/** **kg)**	7.2 ± 0.6***	2.9 ± 0.06***
**Iso** + **tac** ** (2 mg/** **kg)**	4.2 ± 0.5***	2.5 ± 0.09***

^a^P < 0.001, ^c^P < 0.01 from respective control value; *P < 0.05, **P < 0.01 and ***P < 0.001 as compared with ISO-treated group. Data are expressed as mean ± SEM (n = 6).

### Histopathological examination of the cardiac tissue

**Figure 5:**
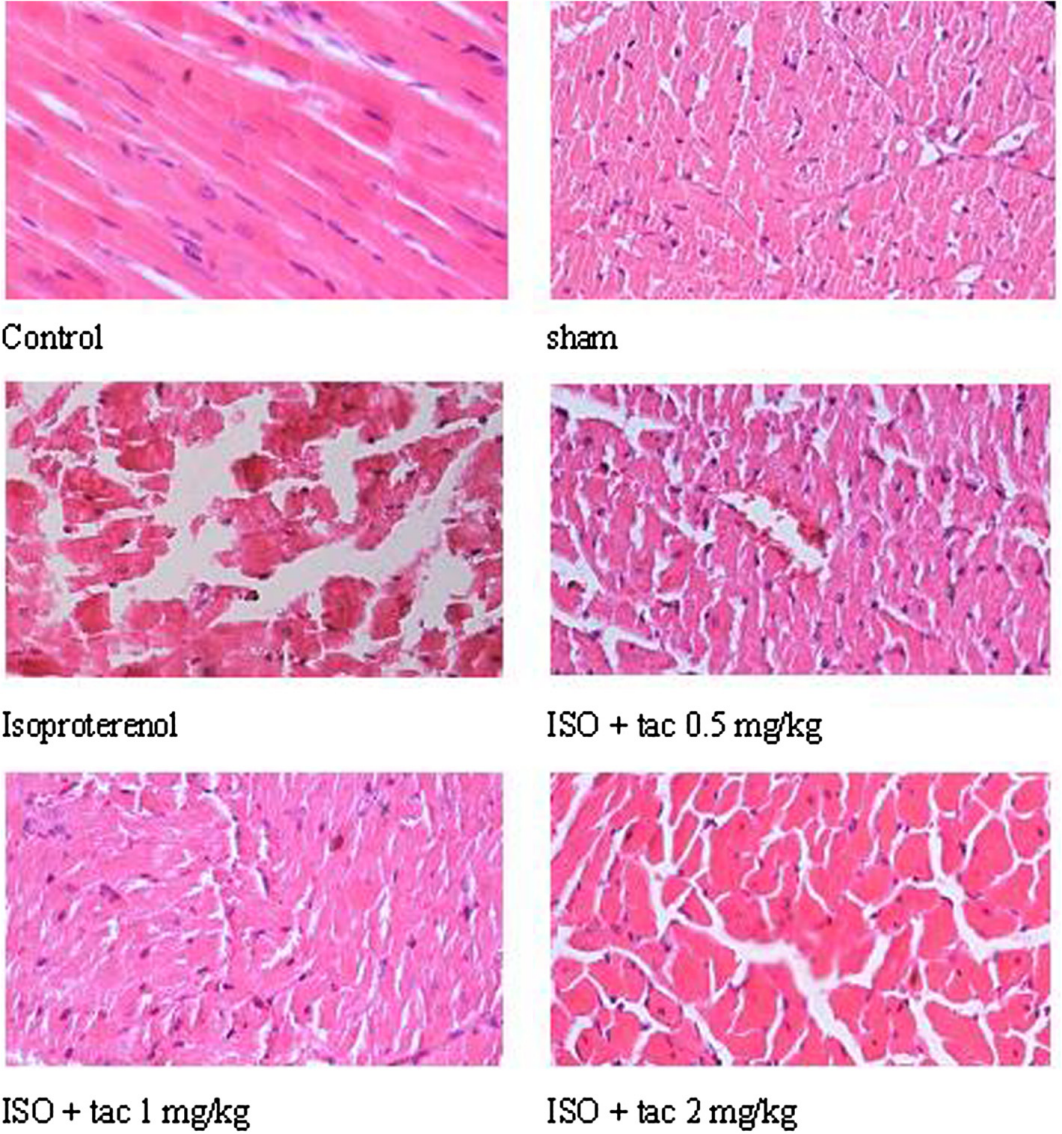
**Photomicrographs of sections of rat cardiac apexes.** Heart tissue of a rat treated with isoproterenol (ISO) showed intensive cardiomyocyte necrosis and increased edematous intramuscular space. Acute treatment with tacrolimus (tac) demonstrated a marked improvement. H&E (40' magnification).

**Figure 6:**
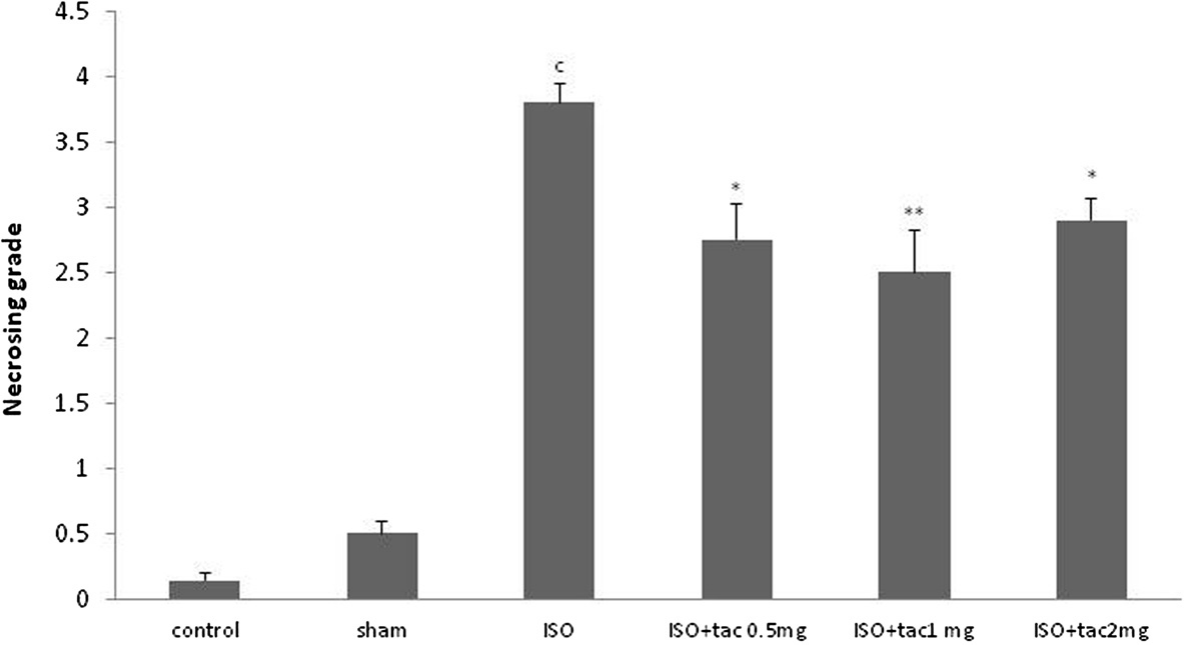
**Grading of histopathological changes in the rat**’**s cardiac apex tissues.** Grades 1, 2, 3, and 4 showed low, moderate, high and intensive pathological changes, respectively. ^c^P < 0.001 compared to respective group in healthy animals, *P < 0.05 and **P <0.01 as compared to isoproterenol treated group. Data are reported as mean ± SEM (n = 6).

In the control group, myocardial fibers were arranged regularly with clear striations, without any damage or necrosis in the tissue (Figure 5). Histopathological sections of the isoproterenol alone treated hearts displayed extensive hypertrophy, necrosis and increased edematous inter muscular space. Scoring the necrosis showed that all three doses of tacrolimus significantly prevented inflammatory responses. Tacrolimus at doses of 0.5, 1 and 2 mg/kg reduced isoproterenol-induced edema and necrosis by 28% (P < 0.05), 35% (P < 0.01) and 24% (P < 0.05), respectively (Figure 6).

### Effects of tacrolimus on the level of TNF-α in serum after myocardial infarction

**Figure 7:**
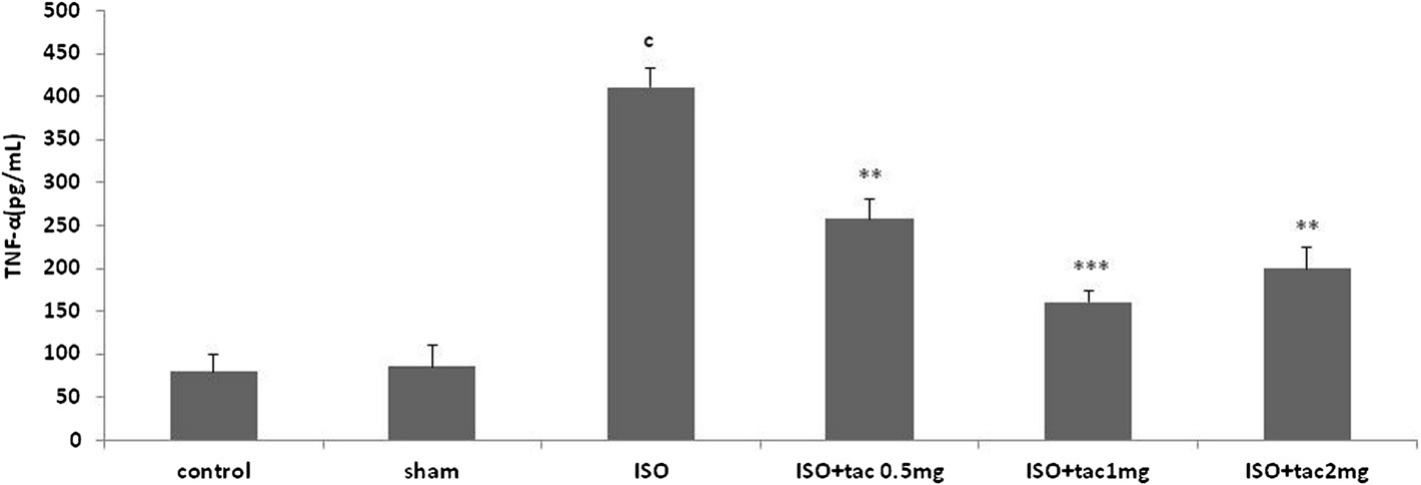
**The effect of acute and short term treatment with tacrolimus** **(tac)** **at the doses of 0.5,** **1 and 2 mg/** **kg/** **12 h on TNFα concentration in serum.** ^c^P < 0.001 from respective control value; **P < 0.01 and ***P < 0.001 as compared with isoproterenol (ISO) injected group. Data are reported as mean ± SEM (n = 6).

In the present study, the effects of tacrolimus on the concentration of TNF-α in serum of rats with isoproterenol induced MI were also investigated in order to determine whether inflammatory condition in myocardium are associated with the level of pro-inflammatory cytokines. It was found that the level of TNF-α was profoundly increased in the isoproterenol group in comparison to the control group (P < 0.001) in serum (Figure 7). It was observed that tacrolimus alone (100 mg/kg/12 h) in the sham group had no effect on the level of TNF-α in serum. The increased level of TNF-α by isoproterenol was markedly decreased by treatment with tacrolimus serum. The maximal effect was seen by 1 mg/kg of tacrolimus, where the concentration of TNF-α was significantly reduced from 410 ± 22 pg/mL of serum to 160 ± 14 pg/mL (P < 0.001) (Figure 7).

## Discussion

Electrocardiogram (ECG) is considered the most important clinical tool for the diagnosis of many types of myocardial infarction, especially for detection of ST segment elevation myocardial infarction (STEMI). Subcutaneous injection of isoproterenol (100 mg/kg) for two consecutive days caused ST- segment elevation and R-amplitude depression. The elevated ST-segment implies the loss of cell membrane function and the consequent potential difference between the ischemic and non-ischemic regions and the depressed R-amplitude might be due to the isoproterenol-induced myocardial edema [14]. Tacrolimus administration significantly amended the ECG pattern, indicating its protective effects on cell membrane function and electrical discharges.

Isoproterenol injection was followed by a significant decrease in arterial pressure indices, including ventricular contractility (LVdP/dt_max_) and relaxation (LVdP/dt_min_), as well as an increase in the left ventricular end-diastolic pressure (LVEDP). These disturbances were compatible with the hemodynamic changes occurring in the acute myocardial infarction.

The principal goal of post-MI therapy is to preserve the contractility of the heart (LVdP/dt_max_ and LVdP/dt_min_) and to decrease the elevated LVEDP. Lower values of LVEDP result in improved blood flow to the subendocardial tissue, which is greatly susceptible to ischemic necrosis.

The results of the present study clearly demonstrated the beneficial effects of acute treatment with tacrolimus, administered concurrently with the induction of myocardial infarction, on various parameters of cardiac function. Tacrolimus was found to effectively raise the arterial blood pressure and myocardial contractility, along with decreasing the left ventricular end diastolic pressure.

Tacrolimus had more significant protective effects on the left ventricular function at the low dose of 1 mg/kg compared with the dose of 2 mg/kg. This might be due to the paradoxical effects of the high dose of tacrolimus on inflammatory process and on intracellular calcium homestasis. Tacrolimus binds to FKBP and suppresses inflammatory responses. On the other hand, since FKBP plays a critical role in modulating myocardial calcium homeostasis by stabilizing ryanodine receptors, the binding may disturbs the calcium homeostasis and therefore, reverse the protective effects of the drug [7,11]. It seems that the high dose of the drug reduces remodeling by suppressing inflammatory responses and meanwhile, it inhibits the stabilizing effects of FKBP on ryanodine receptors by binding to it [17].

The importance of oxidative stress is implicated in the pathology of myocardial infarction. There is appreciable evidence validating the direct toxic effects of free radicals on heart tissue [18]. In fact, one of the mechanisms of isoproterenol-induced myocardial infarction is to generate highly cytotoxic free radicals, as superoxide. Malondialdehyde, a product of the oxidative degradation of unsaturated fatty acids, is a biomarker of oxidative stress, and the concentration increases in response to the free radical production in myocardial infarction, and decreases by antioxidant systems [19,20]. The results of the present study suggest that tacrolimus possesses an in vivo antioxidant activity. Tacrolimus administration substantially reduced peroxylipid levels by 50 - 70% in isoproterenol-induced myocardial infarction. This reduction may relate to blunted polymorphonuclear leukocyte accumulation [21]. Tacrolimus treatment ameliorates oxidative stress augmentation which plays a pivotal role in the myocardial infarction pathology.

Histopathological examination of infarcted myocardial tissue elucidated extensive hypertrophy, subendocardial necrosis, and edema. All three doses of tacrolimus considerably attenuated the edema and necrosis in the isoproterenol-treated heart.

The heart to body weight ratio was significantly increased following isoproterenol injection. The heart weight gain might be due to water absorption and increase in protein content of muscular cells. Cardiac hypertrophy is due to increased cardiac performance. Treatment with all doses of tacrolimus markedly reduced the heart to body weight ratio. However, the drug was very effective at the low dose of 1 mg/kg.

## Conclusions

Limited data are available on the cardioprotective effects of tacrolimus. However, our study finds that administration of tacrolimus, simultaneous to the myocardial infarction incidence, has a remarkable protective potential against damages caused by isoproterenol-induced myocardial infarction. The cardioprotective effects of acute treatment with tacrolimus are confirmed by amending electrocardiographic pattern, improvement of hemodynamic and left ventricular functions, and less histopathological damage following isoproterenol-induced myocardial infarction. It is proposed that tacrolimus exerts this cardioprotective effects through its anti-inflammatory and antioxidant properties. Although this study has provided a possible new therapeutic tool for myocardial infarction, more studies are required to elucidate the precise mechanism of tacrolimus in reversing the pathogenesis of myocardial infarction.
